# Chemical and Nutritional Fat Profile of *Acheta domesticus*, *Gryllus bimaculatus*, *Tenebrio molitor* and *Rhynchophorus ferrugineus*

**DOI:** 10.3390/foods13010032

**Published:** 2023-12-21

**Authors:** Agnieszka Orkusz, Lucyna Dymińska, Karol Banaś, Joanna Harasym

**Affiliations:** 1 Department of Biotechnology and Food Analysis, Wroclaw University of Economics and Business, 53-345 Wroclaw, Poland; karol.banas@ue.wroc.pl (K.B.); joanna.harasym@ue.wroc.pl (J.H.); 2 Department of Bioorganic Chemistry, Wroclaw University of Economics and Business, 53-345 Wroclaw, Poland; lucyna.dyminska@ue.wroc.pl

**Keywords:** edible insects, fat, fatty acids, chemical composition, nutritional standards

## Abstract

The use of edible insects in the human diet is gaining importance because they are characterized by high nutritional value, and their cultivation is much more environmentally friendly than traditional livestock farming. The objective of this study was to determine the chemical and nutritional fat profile of selected edible insects as follows: house cricket (*Acheta domesticus* adult), field cricket (*Gryllus bimaculatus* adult), mealworm (*Tenebrio molitor* larvae), and palm weevil (*Rhynchophorus ferrugineus* larvae) which are now commercially available worldwide. Additionally, the degree of implementation of nutrition standards for selected nutrients by these insects was assessed. Freeze-dried insects were studied using infrared-attenuated total reflectance mid-infrared spectroscopy for basic differentiation. The content of fat and fatty acids was determined, and dietary indicators were calculated. The spectroscopic findings align with biochemical data, revealing that *Rhynchophorus ferrugineus* larvae contain the highest fat content and the least protein. Unsaturated fatty acids (UFAs) predominated in the fat of the assessed insects. The highest content of saturated fatty acids (SFAs), along with the lowest content of polyunsaturated fatty acids (PUFAs), was observed in the larvae of the *Rhynchophorus ferrugineus* species. From a nutritional standpoint, *Tenebrio molitor* larvae exhibit the most favorable indicators, characterized by minimal athero- and thrombogenic effects, along with an optimal balance of hypo- and hypercholesterolemic acids. Knowledge of the composition and quantities of fats in different insect species is valuable for planning and preparing meals with accurate nutritional profiles, among other applications.

## 1. Introduction

The nutritional value of a product is primarily determined by its chemical composition and the content of exogenous ingredients. It is widely known that edible insects are characterized by high nutritional value, influenced by their high protein content [1,2]. In addition, insects are a good source of vitamins (A, B_6_, and B_12_) and minerals (Fe, Ca, Zn, and Se) [2,3]. They can, therefore, serve as an alternative to traditional protein sources, especially meat [2,4]. There is a belief [5,6] that meat is a significant and essential element of a well-balanced diet in highly developed societies, ensuring optimal growth and development of the body. Throughout evolution, humans have adapted to a diet rich in large quantities of meat. However, due to the increasingly common occurrence of diet-related civilization diseases, significant importance is being placed on the quality of products consumed by humans. For the future of the Earth and the next generations, there is a need for a crucial shift in eating habits and new food products based on organic production systems.

The interest in insects as a food source is primarily due to two issues. Firstly, they are perceived as a sustainable protein source for future food demands, and secondly, they can counteract malnutrition, especially in developing countries [7].

Insect farming is more environmentally sustainable compared to traditional animal farming. It requires less space, water, and feed. Insects can be fed with organic waste. Additionally, efficiently converting feed into protein is connected with fewer greenhouse gases [7]. Insects, being cold-blooded animals, can be euthanized humanely by cooling them. This process ensures their demise without pain, suffering, or stress.

Throughout history, people globally have incorporated insects into their daily meals for thousands of years. This practice has been prevalent in Africa, Asia, Australia, and the Americas since ancient times and continues to persist today [7,8].

Over 2000 edible insect species are consumed worldwide [9]. Predominantly, beetles, caterpillars, bees, wasps, ants, grasshoppers, locusts, crickets, true bugs, dragonflies, termites, flies, cockroaches, and various other orders constitute the most frequently consumed groups of insects. Insects are eaten at various life cycle stages (e.g., eggs, larvae, pupae, and adults) [10]. In Asia, red ant eggs [11], wasp and honeybee larvae, and the pupae of weaver ants are popular. Palm beetle larvae are considered a delicacy in Latin America, Asia, and Africa. Crickets and grasshoppers are favored both in Africa and in Asia. In Australia, Aborigines eat moths. Caterpillars are widely consumed in central and southern Africa [10].

The surge of interest in entomophagy is becoming increasingly prominent in Western societies. The United Nations Food and Agriculture Organization (FAO) has played an essential role in promoting the integration of edible insects into the human diet. In 2013, FAO published an extensive report [7] indicating the arguments supporting Westerners’ adoption of this novel food source. The formal recognition of whole insects and their constituent parts as novel foods occurred through Regulation 2015/2283 enacted by the European Parliament and the Council of the European Union [12]. Moreover, in 2015, the European Food Safety Authority (EFSA) presented a list of insect species deemed to have substantial potential as food for humans and animals [13]. In 2021, the EFSA issued positive scientific opinions on the safety of dried yellow mealworm (*Tenebrio molitor* larva) [14], frozen and dried migratory locusts (*Locusta migratoria*) [15], and whole crickets (*Acheta domesticus* larva) [16], and in 2022 on freeze-dried preparations of the lesser mealworm (*Alphitobius diaperinus* larva) as a novel food [17] according to Regulation (EU) 2015/2283. Within the European Union, approval for marketing is presently granted to only four edible insect species: *Tenebrio molitor* larva (EU Regulation 2021/882 and 2022/169) [18,19], *Locusta migratoria* (EU Regulation 2021/1975) [20], *Acheta domesticus* (EU Regulation 2022/188 and 2023/5) [21,22], and *Alphitobius diaperinus* larva (EU Regulation 2023/58) [23].

In addition to the benefits of including edible insects in the human diet, it should be noted that they may cause allergic reactions in people allergic to crustaceans, mollusks, and mites. Additionally, insects may contain additional allergens if they are present in their food [18,19,20,21,22,23].

This study aimed to determine the chemical and nutritional fat profile of selected edible insects as follows: house cricket (*Acheta domesticus* adult), field cricket (*Gryllus bimaculatus* adult), mealworm (*Tenebrio* molitor larvae), and palm weevil (*Rhynchophorus ferrugineus* larvae) and assessment of the degree of implementation of nutrition standards for selected nutrients by these insects.

## 2. Materials and Methods

### 2.1. Research Material

The research material included four types of powdered freeze-dried insects: *Acheta domesticus* (adult)*, Gryllus bimaculatus* (adult), *Tenebrio molitor* (larvae), and *Rhynchophorus ferrugineus* (larvae). The insects were purchased commercially. These are species cultured on a large scale and sold commercially for use as food and feed [24,25,26,27].

### 2.2. Infrared Measurements

FTIR/ATR spectra were captured within the 300–4000 cm^−1^ range using a Nicolet 6700 spectrometer (Thermo Fisher Scientific, Waltham, MA, USA) equipped with a portable ATR set. The measurements were conducted with a resolution of 2 cm^−1^. All samples underwent measurement under uniform conditions to facilitate a comparative analysis of their vibrational spectra [28].

### 2.3. Fat Determination and Gas Chromatographic Analysis

The fat concentration was determined following the AOAC Official Method 991.36 (AOAC, 1990) [29].

For the determination of fatty acids (FAs) composition, the lipid samples were converted to their corresponding methyl esters by AOCS Official Method Ce 2-66 [30]. The FA methyl esters were quantified by gas chromatography using Perkin Elmer Clarus 580 (Perkin Elmer Inc., Waltham, MA, USA) gas chromatograph equipped with a silica capillary column with Rtx2330 stationary phase, with a length of 105 m and flame-ionization detector (GC-FID). Hydrogen was used as the carrier gas at a head pressure of 1.5 mL/min constant flow. The temperature of the detector and injector was 240 °C. The initial column temperature was set at 165 °C for 10 min, then raised to 220 °C at a rate of 2 °C/min. Fatty acids were presented as a percentage of the total amount of the methyl esters.

### 2.4. Dietary Indicators

Atherogenic indexes (AI), thrombogenic index (TI) [31], and hypocholesterolemic/hypercholesterolemic ratio (h/H) [32,33] were calculated according to the following equations:

AI = (C12:0 + 4 × C14:0 + C16:0)/(MUFA + *n* − 6 + *n* − 3);

TI = (C14:0 +C16:0 + C18:0)/(0.5 × MUFA+ 0.5 × *n* − 6 + 3 × *n* − 3 + *n* − 3/n − 6);

h/H = (C18:1 *c*9 + C18:2 *n* − 6 + C18:3 *n* − 3 + C20:3 *n* − 6 + C20:4 *n* − 6 + C20:5 *n* − 3 + C22:5 *n* − 3)/(C12:0 +  C14:0 + C16:0).

where MUFA—monounsaturated fatty acids.

### 2.5. Reference Values

The reference values for protein (%), fiber (%), and energy value (kcal/100 g) per 100 g of edible were provided based on the manufacturer’s nutrition declaration included on the product label. Following the applicable legal provisions, i.e., Regulation (EU) No. 1169/2011 of the European Parliament and the Council on the provision of food information to consumers [34], one of the mandatory information that must be provided on food packaging is the nutritional value (called ‘nutrition declaration’), expressed in per 100 g or 100 mL of food product. Mandatory elements provided in nutritional information are energy value and the content of fat, saturated fatty acids, carbohydrates, sugars, protein, and salt. Additionally, food producers may extend it to include the content of mono- and polyunsaturated fatty acids, polyhydric alcohols, starch, dietary fiber, and vitamins. [34]. The fat and fatty acids profile was given following the determination made in Section 2.3.

### 2.6. Statistical Analysis

The data were processed statistically [35] by calculating arithmetic means and standard deviations. All analyses were performed in triplicate. One-way variance analysis (ANOVA) was used to evaluate differences between the analyzed parameters (fatty acids, dietary indicators). When a significant effect was identified, the mean values were further analyzed using Tukey’s multiple range test. Differences with a probability level < 0.05 were regarded as statistically significant.

### 2.7. Ethical Statement

Ethical review and approval were waived for this study due to the research being based on the food products that are commercially available on the market.

## 3. Results and Discussion

Infrared spectroscopy is an excellent method to study the structure and properties of biological systems. Infrared spectra of the analyzed samples were recorded in the range 4000–400 cm^−1^. The compared spectra of the studied samples differed in the absorbance strength and peak position, which is related to changes in the chemical composition of the insects. To compare the quantitative content of the individual components of studied samples, the measured spectra should be standardized; therefore, the common point of the spectra was determined, in this case, the maximum intensity of the band at 2850 cm^−1^, corresponding to νs(CH_2_) vibrations.

In the FTIR spectra of all samples, the broad band at 3700–3000 cm^−1^ corresponds to the stretching ν(OH) and ν(NH_2_) modes of the free hydroxyl and amine groups and those involved in the hydrogen bonds. The stretching of O-H bonds mostly comes from carbohydrates, mainly from chitin. Moreover, all these spectra exhibited two narrower IR bands at around 2900 and 2850 cm^−1^, characteristic of asymmetrical and symmetrical stretching vibrations from C-H bonds from methylene groups presumably caused by lipids or, in the case of insect powders, lipids, and chitin.

Another typical IR band between all the ingredients was observed around 1740 cm^−1^. This IR band can be associated with stretching C=O bonds from ester groups related to lipids.

A few FTIR spectra regions were usually studied to characterize the structures of different proteins and amino acids. For example, Amide A (3225–3280 cm^−1^) is due to the N-H stretching vibration. The principal Amide I (1700–1600 cm^−1^) and Amide II (1600–1500 cm^−1^) regions are mainly associated with the stretching vibrations of peptide carbonyl groups. The bands from the range 1460–1100 cm^−1^ can be assigned to the vibration of lipids or carbohydrates. Finally, a distinctive IR band at 1100–900 cm^−1^ corresponds to C-O stretching vibrations, probably from carbohydrates, such as chitin [36].

The FTIR spectrum of chitin was previously analyzed in literature [37,38]. Based on these data, the dominant absorption bands can be assigned: 3450 cm^−1^—ν(OH)HB, 3106 cm^−1^—νs(NH), 2930 cm^−1^—νas(CH_3_), 2890 cm^−1^—νs(CH_3_), 1663 cm^−1^—amide I: ν(C=O), 1627 cm^−1^—δ(NH), 1560 cm^−1^—amide II: ν(CN) + δ(NH,), 1415 cm^−1^—δs(CH_3_) + δ(CH), 1379 cm^−1^—δ(CH) + δ(C-CH_3_), 1316 cm^−1^—amide III: ν(CN) + δ(NH), 1262 cm^−1^—δ(NH), 1158 and 1116 cm^−1^—νas(COC) + ν(ϕ), 1074 and 1027 cm^−1^—ν(C-O), 951 cm^−1^—ν(CN), 896 cm^−1^—ρ(CH_2_), 703 cm^−1^—amide V: ω(NH) + δ(ϕ) and 690 cm^−1^—δ(ϕ).

**Figure 1:**
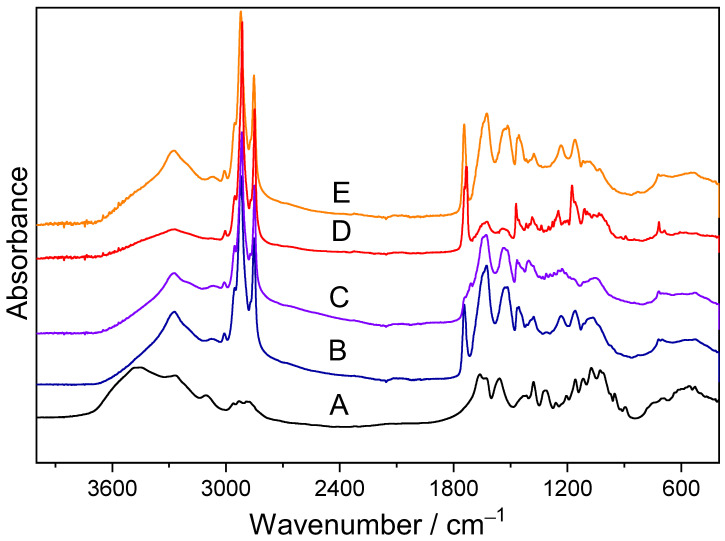
FTIR spectra of chitin (**A**) and *Acheta domesticus* (**B**), *Gryllus bimaculatus* (**C**), *Rhynchophorus ferrugineus* (**D**), and *Tenebrio molitor* (**E**).

The spectra of analyzed samples (Figure 1) clearly show great similarities to the spectrum of chitin. The FTIR spectra of studied samples are similar in both the position and intensity of the strongest bands. However, these spectra contain several additional bands corresponding to other sample ingredients.

In order to compare the content of lipids, proteins, and chitin between the tested samples, selected broad contours were deconvoluted into Lorentz components, and the integral intensities of individual components were compared.

The integral intensities (I) of bands characteristic of the chitin vibrations, observed in the IR spectra of studied samples [*Acheta domesticus* (1), *Gryllus bimaculatus* (2), *Tenebrio molitor* (3), and *Rhynchophorus ferrugineus* (4)] show the following relationships:

- For the band at 3450 cm^−1^: I2 > I1 ≥ I3 > I4;
- For the band at 3106 cm^−1^: I2 > I1 ≥ I3 > I4;
- For the band at 1663 cm^−1^: I1 ≥ I3 > I2 > I4;
- For the band at 1116 cm^−1^: I1 > I4 > I2 ≥ I4;
- For the band at 1074 cm^−1^: I1 > I2 > I3 > I4;
- For the band at 1027 cm^−1^: I1 > I2 > I3 > I4.

These data suggest that palm weevil larvae contain a smaller amount of chitin than other samples.

In the infrared spectra of the studied samples, there are bands characteristic of lipid vibrations. There are mainly bands at the following wavenumbers: 3007 w—νas(=C-H), 2952 m—νas(CH_3_), 2922 vs—νas(CH_2_), 2871 s—νs(CH_3_), 2854 s—νs(CH_2_), 1744 vs—ν(C=O), 1462 m—δas(CH_3_) + δas(CH_2_), 1173 m—ν(C−O), and 716 m cm^−1^—γ(C-C-C) (where vs is a band with very strong intensity; s—a band with a strong intensity; m—medium intensity band; and w—low-intensity band) [28]. Vibrational analysis indicates a higher lipid content in palm weevil larvae relative to other ingredients (Ilipids > Iproteins, carbohydrates) compared to house cricket, field cricket, and mealworm. This is evidenced by sharp bands of high intensity at the following wavenumbers: 1462, 1173, and 716 cm^−1^. In addition, a double band is observed: 1744 and 1732 cm^−1^, which indicates the presence of C=O bonds in free carboxyl groups and those involved in interactions. *Gryllus bimaculatus* is marked with a lower lipid content than other ingredients in this sample (Iproteins, carbohydrates > Ilipids). The intensities of the bands characteristic of lipid vibrations are weak and/or included in the broader bands characteristic of vibrations of other ingredients, e.g., carbohydrates and proteins. The bands corresponding to lipid vibrations in the spectra of *Acheta domesticus* and *Tenebrio molitor* have similar locations and intensities. However, it can be noticed that in the house cricket, intensities of these bands are lower than those characteristic of vibrations of other ingredients (Iproteins, carbohydrates > Ilipids).

The integral intensities of the Lorentz components characteristic of lipids vibrations observed for palm weevil larvae are higher than those of other insects, respectively. The integral intensities of these bands for the studied samples fulfill the following dependences:

- For the band at 3007 cm^−1^: I4 > I3 > I1 ≥ I2;
- For the band at 1740 cm^−1^: I4 > I3 ≥ I1 > I2;
- For the band at 1173 cm^−1^: I4 > I3 > I1 ≥ I2;
- For the band at 716 cm^−1^: I4 > I3 > I1 ≥ I2.

House cricket (*Acheta domesticus*), field cricket (*Gryllus bimaculatus*), and mealworm (*Tenebrio molitor*) have the highest protein content compared to the other ingredients (Iproteins > Ilipids, carbohydrates). This is indicated by wide, intense bands termed amide A (3278 cm^−1^), amide I (1644, 1623 cm^−1^), and amide II (1533, 1513 cm^−1^). The most intense amide I band corresponding to C=O stretching and NH bending vibrations of the amide group is particularly useful for protein analyses. The amide II band can be associated with NH bending and CN stretching vibrations of the amide group. Amide A band is characteristic of NH stretching vibrations coupled with amide II overtone [39,40].

The integral intensities of these bands characteristic of protein vibrations for the studied samples fulfill the following dependences:

- For the band at 3278 cm^−1^: I3 > I1 > I2 > I4;
- For the band at 1664 cm^−1^: I1 > I3 > I2 > I4;
- For the band at 1623 cm^−1^: I1 > I3 > I2 > I4;
- For the band at 1533 cm^−1^: I2 > I3 > I1 > I4;
- For the band at 1513 cm^−1^: I1 > I2 > I3 > I4.

The spectroscopic analysis of the composition of insects was consistent with the biochemical data obtained using reference methods (Table 1). Compared to other insect species, larvae of the *Rhynchophorus ferrugineus* species contained the highest amount of fat and the least amount of protein. At the same time, a similar protein content was demonstrated in *Acheta domesticus, Gryllus bimaculatus*, and *Tenebrio molitor.*

The biological value of fat is primarily determined by the quantity and type of fatty acids present in it. Although the content of fatty acids varied depending on the insect species, it was demonstrated that unsaturated fatty acids predominated in the fat of the assessed insects (Table 2). The results are consistent with the literature data because insects usually contain more unsaturated fatty acid (UFA) than SFA [41]. The highest content of SFA, along with the lowest content of PUFA, was observed in the larvae of the *Rhynchophorus ferrugineus* species. Regardless of the insect species, among the saturated fatty acids (Table 2), the highest share has palmitic acid (C 16:0) and is followed by stearic acid (C 18:0). Oleic acid (C 18:1) stands out as the predominant monounsaturated fatty acid.

Of particular importance for the human body is the presence of polyunsaturated fatty acids (linoleic—C 18:2 *n −* 6 and linolenic C 18:3 *n −* 3 acids), classified as essential fatty acids that must be supplied with food because they are not produced in the body. *Acheta domesticus* has the highest content of linoleic acid compared to other insect species. In turn, *Rhynchophorus ferrugineus* has the highest content of linolenic acid and, concurrently, the lowest content of linoleic acid among the studied insect species.

The ratio of polyunsaturated to saturated fatty acids serves as a crucial indicator of fat quality, with a recommended dietary amount exceeding 0.40 [42]. Consequently, in terms of human nutrition, the fat from insect species like *Tenebrio molitor*, *Acheta domesticus*, and *Gryllus bimaculatus* demonstrates a more favorable PUFA/SFA ratio than *Rhynchophorus ferrugineus.* The fat content and fatty acid profile of the tested insects were similar to the results of other studies [43,44,45].

The results showed the content of individual nutrients varies. This variation was expected because the nutritional value of insects depends on their developmental stage [46], diet [45,47,48], sex [45,49], season [45], and the way insects are prepared and processed before consumption [50]. The influence of diet on the fatty acid profile of insects is also known [45], as it likely mirrors the fatty acid composition of their feed. Thus, insects’ nutritional content originating from the same producer may exhibit variations {45]. Hence, the industry needs to emphasize the adoption of a standardized rearing protocol, a key measure to uphold the uniformity of nutritional profiles across diverse production batches [51].

Fatty acid indexes, specifically AI, TI, and h/H, provide valuable insights into the potential impact of ingested lipids, offering a more comprehensive evaluation of the nutritional quality of food compared to a simple consideration of SFA, MUFA, PUFA, and their ratios. The atherogenicity index highlights the association between pro-atherogenic saturated fatty acids, which promote lipid attachment to endothelial cells, and antiatherogenic unsaturated fatty acids, which reduce cholesterol levels and prevent coronary artery diseases. The thrombogenicity index assesses the tendency for blood clot formation in vessels, as indicated by the ratio of saturated to unsaturated fatty acids, albeit in varying proportions. From a nutritional standpoint, *Tenebrio molitor* larvae exhibit the most favorable indicators, characterized by minimal athero- and thrombogenic effects, along with an optimal balance of hypo- and hypercholesterolemic acids (Table 2).

Nutritional recommendations for adults regarding the percentage of energy obtained from protein and fat, including SFA, MUFA, and PUFA acids, are presented in Table 3.

For a diet requiring 2000 kcal, the intake of 100 g of edible insects per day covered (depending on the insect species) from 32.8% to 81.0% of the recommended SFA intake, from 14.6% to 48.2% MUFA, from 29.0% to 60.5% PUFA, and 29.3–134.1% and 4.5–40.0% for *n −* 6 PUFA and *n −* 3 PUFA, respectively. The analyzed species of edible insects were a significant source of fat, covering the daily requirement for this ingredient in adults, ranging from 29.3% to 92.8%. At the same time, a serving of insects (100 g) constituted a significant source of protein, covering the daily requirements for this component in adults ranging from 34.4% to 113.6% (Table 3).

Available evidence and dietary recommendations show that there is no association of total fat, monounsaturated fatty acid (MUFA), polyunsaturated fatty acid (PUFA), and saturated fatty acid (SFA) with the risk of chronic diseases. The fact is that SFA replacement with PUFA and/or MUFA improves blood lipids and glycemic control, with the effect of PUFA being more pronounced [54]. Overall, the available published evidence deems it reasonable to recommend the replacement of SFA with MUFA and PUFA [54,55]. Therefore, from a nutritional point of view, palm weevil larvae had the least favorable composition of fatty acids and dietary indicators.

Although insects are generally considered primarily a source of protein, as the results of our research indicate, they also are a source of nutritionally valuable fat. They can be used, among others, to improve the texture and taste of food products. However, one can know that insects rich in unsaturated fatty acids are susceptible to oxidation. Lipid oxidation byproducts generated during the production, processing, and storage of insect-containing products may adversely affect their physicochemical properties, leading to a deterioration in their overall quality.

## 4. Conclusions

Analyzing the spectral composition of insects was consistent with the biochemical data. Fat constituted the second most significant nutritional component in edible insects, closely trailing behind protein. The analyzed species of edible insects were a significant source of fat, covering the daily requirement for this ingredient in adults, ranging from 29.3% to 92.8%. Among studied insect species, the larvae of *Rhynchophorus ferrugineus* exhibited the highest fat content and the lowest protein levels. *Acheta domesticus, Gryllus bimaculatus*, and *Tenebrio molitor* displayed comparable fat content.

As a sustainable protein and fat source, edible insects present a viable option for diversifying diets and meeting nutritional needs. Integrating edible insects into a diet can improve its nutritional value, especially with species such as *Tenebrio molitor*, *Acheta domesticus*, and *Gryllus bimaculatus.* These insects present favorable fatty acid profiles, delivering crucial nutrients (polyunsaturated fatty acids) while mitigating athero- and thrombogenic effects. Consequently, they can be utilized as a source of essential fatty acids instead of conventional edible oils for human nutrition.

Understanding the composition of nutrients and their content in different insect species can assist in the thoughtful curation and preparation of meals, ensuring an appropriate balance of essential elements. This awareness has the potential to influence dietary choices, prompting a shift in eating patterns and contributing to an overall enhancement in human well-being.

## Figures and Tables

**Table 1:** Reference values of basic composition (%) and energy value (kcal/100 g) per 100 g of edible insects.

Parameter	*Acheta* *domesticus* (A)	*Gryllus* *bimaculatus* (A)	*Tenobrio* *molitor* (L)	*Rhynchophorus* *ferrugineus* (L)
Protein	56.8	53.4	56.0	25.8
Fat	22.8	26.4	26.6	40.8
Fibre	3.3	6.6	5.3	* ns
Energy	518.0	466.7	511.0	583.0

* ns—not specified. A—adult insect; L—larval form.

**Table 2:** Fatty acids content and dietary indicators in edible insects [%].

Fatty Acids and Dietary Indicators	*Acheta* *domesticus* (A)	*Gryllus bimaculatus* (A)	*Tenobrio* *molitor* (L)	*Rhynchophorus ferrugineus* (L)
SFA	37.29 ^b^ ± 1.70	35.13 ^b^ ± 1.86	27.06 ^a^ ± 1.44	43.60 ^c^ ± 3.56
C 12:0	-	0.60 ± 0.03	-	-
C 14:0	0.44 ^a^ ± 0.02	0.91 ^b^ ± 0.05	2.97 ^c^ ± 0.16	2.89 ^c^ ± 0.24
C 16:0	25.02 ^b^ ± 1.14	25.46 ^b^ ± 1.35	18.99 ^a^ ± 1.01	34.49 ^c^ ± 2.82
C 17:0	-	-	0.19 ± 0.01	0.17 ± 0.01
C 18:0	10.69 ^d^ ± 0.49	7.06 ^c^ ± 0.37	3.87 ^a^ ± 0.21	5.26 ^b^ ± 0.43
C 20:0	0.61 ^c^ ± 0.03	0.53 ^b^ ± 0.03	0.41 ^a^ ± 0.02	0.39 ^a^ ± 0.03
C 22:0	0.22 ^b^ ± 0.01	-	-	0.15 ^a^ ± 0.01
MUFA	28.22 ^a^ ± 1.29	38.57 ^b^ ± 2.04	46.13 ^c^ ± 2.46	38.92 ^b^ ± 3.18
C 16:1	0.57 ^a^ ± 0.03	2.30 ^c^ ± 0.12	1.46 ^b^ ± 0.08	2.40 ^c^ ± 0.20
C 18:1	24.28 ^a^ ± 1.11	32.15 ^b^ ± 1.70	40.20 ^c^ ± 2.14	32.36 ^b^ ± 2.64
C 24:1	-	0.26 ± 0.01	0.23 ± 0.01	-
PUFA	34.49 ^c^ ± 1.57	26.29 ^b^ ± 1.39	26.76 ^b^ ± 1.43	17.48 ^a^ ± 1.43
C 18:2 *n −* 6	32.82 ^c^ ± 1.50	24.29 ^b^ ± 1.29	25.41 ^b^±1.35	16.18 ^a^ ± 1.32
C 18:3 *n −* 3	0.88 ^b^ ± 0.04	0.91 ^bc^ ± 0.05	0.68 ^a^ ± 0.04	1.05 ^c^ ± 0.09
UFA	62.71 ^a^ ± 2.86	64.83 ^ab^ ± 3.43	72.94 ^b^ ± 3.89	56.40 ^a^ ± 4.61
PUFA/SFA	0.92 ^c^ ± 0.04	0.75 ^b^ ± 0.02	0.99 ^c^ ± 0.05	0.40 ^a^ ± 0.02
PUFA *n −* 3	1.10 ^b^ ± 0.05	1.13 ^b^ ± 0.06	0.75 ^a^ ± 0.04	1.08 ^b^ ± 0.09
PUFA *n −* 6	32.91 ^c^ ± 1.50	24.33 ^b^ ± 1.29	25.41 ^b^ ± 1.35	16.18 ^a^ ± 1.32
PUFA *n −* 6/PUFA *n −* 3	29.91 ^c^ ± 0.56	21.53 ^b^ ± 0.44	33.88 ^d^ ± 0.62	14.98 ^a^ ± 0.35
AI	0.43 ^a^ ± 0.00	0.46 ^a^ ± 0.01	0.43 ^a^ ± 0.00	0.82 ^b^ ± 0.01
TI	1.07 ^b^ ± 0.02	0.96 ^b^ ± 0.00	0.68 ^a^ ± 0.00	1.38 ^c^ ± 0.01
h/H	2.28 ^b^ ± 0.01	2.13 ^b^ ± 0.01	3.02 ^c^ ± 0.02	1.33 ^a^ ± 0.01

Means with different letters in the same row differ at *p* ˂ 0.05. SFA—saturated fatty acid, MUFA—monounsaturated fatty acids, PUFA—polyunsaturated fatty acid, UFA—unsaturated fatty acid, AI—atherogenic index, TI—thrombogenic index, h/H—hypocholesterolemic/hypercholesterolemic ratio. A—adult insect; L—larval form.

**Table 3:** Coverage of nutritional standards ^a^ for protein, fat, and fatty acids per 100 g portion of edible insects.

Nutrients	Energy Percentage (%E) from the Diet Recommended by FAO ^b^	g/Day (for a 2000 kcal Diet) ^c^	% Coverage for a 2000 kcal Diet			
			*Acheta domesticus* (A)	*Gryllus bimaculatus* (A)	*Tenobrio* *molitor* (L)	*Rhynchophorus ferrugineus* (L)
Protein	10–15	50–75	75.7–113.6	71.2–106.8	66.7–100.0	34.4–51.6
Fat	20–35	44–78	29.3–51.9	33.9–60.2	34.2–60.5	52.3–92.8
SFA	<10	<22	38.7	42.3	32.80	81.0
MUFA	15–20	33–44	14.6–19.5	23.2–30.9	27.9–37.2	36.1–48.2
PUFA	6–11	13–24	32.8–60.5	29.0–53.5	29.7–54.9	29.8–54.9
*n −* 6	2.5–10	5.6–22	34.1–134.1	29.3–115.0	30.8–120.9	30.0–118.0
*n −* 3	0.5–2	1.1–4.4	5.7–22.7	6.8–27.3	4.5–18.2	10.0–40.0

A—adult insect; L—larval form; ^a^ for adults; ^b^ FAO [52] or in the case of protein WHO/FAO [53]; ^c^ UE [34].

## Data Availability

The data presented in this study are available on request from the corresponding author.
